# Assessment of *HER2* status in breast cancer: overall positivity rate and accuracy by fluorescence in situ hybridization and immunohistochemistry in a single institution over 12 years: a quality control study

**DOI:** 10.1186/1471-2407-13-615

**Published:** 2013-12-30

**Authors:** Zsuzsanna Varga, Aurelia Noske, Constanze Ramach, Barbara Padberg, Holger Moch

**Affiliations:** 1 Institute of Surgical Pathology, University Hospital Zurich, Zurich, Switzerland; 2 Institute of Pathology, County Hospital St. Gallen, St. Gallen, Switzerland; 3 Institute of Pathology, County Hospital Aarau, Aarau, Switzerland

**Keywords:** HER2, Fluorescence in situ hybridization (FISH), Immunohistochemistry, Breast cancer

## Abstract

**Background:**

The gold standard of *HER2* status assessment in breast cancer is still debated. Immunohistochemistry (IHC) and in-situ technology as fluorescent-labeled methodology (FISH) can be influenced by pre-analytical factors, assay-conditions and interpretation of test results. We retrospectively conducted this quality control study and analyzed HER2 test results in breast cancer within the routine diagnostic service in a single institution over a period of 12 years. We addressed the question how stable and concordant IHC and FISH methods are and whether HER2 positivity rate has changed over this period.

**Methods:**

Data of 7714 consecutive HER2-FISH-assays in a period of 12 years (2001–2012) on breast cancer biopsies and excision specimens were retrospectively analyzed. From 2001 to 2004, FISH tests were performed from all cases with IHC score 3+ and 2+ (and in some tumors with IHC score 1+ and 0). From 2005–2010, *HER2* status was only determined by FISH. From 2011–2012, all breast carcinomas were analyzed by both IHC and FISH. Scoring and cut-off-definition were done according to time-current ASCO-CAP and FDA-guidelines.

**Results:**

Between 2001–2004, IHC score 3+ was diagnosed in 22% of cases, 69% of these 3+ cases were amplified by FISH. 6% of IHC score 0/1+ cases were amplified by FISH. There was a mean amplification rate of 15.8% (range 13 -19%) using FISH only *HER2*-assays (2005–2010). Starting 2008, a slight drop in the amplification rate from 17% to 14% was noticed due to the modified ASCO-criteria in 2007. From 2011–2012, 12% of cases were 3+ by IHC, 84% of them were amplified by FISH. Less than 1% of IHC score 0/1+ cases were amplified by FISH. Concordance between FISH and IHC increased from 83% to 97%.

**Conclusions:**

Our quality control study demonstrates that *HER2* positivity rate remained stable by FISH-technology but showed a significant variation by IHC over the analyzed 12 years. Improvement in concordance rate was due to standardization of pre-analytical factors, scoring and interpretation.

## Background

Since the FDA approval of Herceptin, the determination of HER2 status became routine in processing breast cancer specimens [1-3]. HER2 status can be assessed on both surgical specimens and core biopsies using national and international guidelines [3,4]. The FDA approved methodologies include the assessment of the protein level by immunohistochemistry (IHC) or gene copy count on the DNA level by in situ hybridization technology (ISH) [2-5]. Current guidelines give clear specifications as to quality assurance, participation in proficiency testing and defined scoring systems [3,5,6]. Furthermore, ASCO/CAP guidelines include the achievement of a concordance level of at least 95% for both positive and negative tumors when using IHC and ISH technology [3,5,6]. As a result of more than a decade intensive research in HER2 testing in breast cancer, it became apparent that testing results can be crucially influenced by pre-analytical and interpretation issues. Concordance between IHC and ISH results vary from 50-100% in the literature [1,2,6-9]. Economic issues of cost-effectiveness of IHC or ISH as primary test have been the issue of several previous studies and are still debated [10,11].

In this study we retrospectively analyzed *HER2* positivity-rate and accuracy resp. concordance-rate between IHC and FISH technology on a large breast cancer cohort (n = 7714) in an exclusively diagnostic setting over 12 years in a single institution. We addressed the question, what factors contributed to poor, acceptable and excellent performance in HER2 testing and explored the reasons behind them.

We are not aware of any similar studies on systematical analysis of HER2 positivity-rate and accuracy in a solely routine diagnostic setting using three different approaches as IHC primary testing with complimentary FISH assay, FISH tests as primary testing and double testing using IHC and FISH on a large diagnostic breast cancer cohort.

## Methods

### Patient collective

The databank on *HER2* FISH testing in breast cancer of the Institute of Surgical Pathology, University Hospital Zurich, Switzerland was retrospectively analyzed in a 12 years period (2001–2012). Altogether 7389 consecutive breast cancer cases undergoing *HER2* FISH testing in the routine diagnostic service were included in the study.

On primary breast cancers, HER2 status was determined on formalin fixed paraffin embedded tissues including breast core biopsies, vacuum assisted biopsies and/or surgical specimens.

On metastastic lesions, cytology specimens and/or paraffin embedded surgical specimens were submitted to HER2 analysis.

### Diagnostic algorithm for *HER2* testing between 2001 and 2012

2001–2004: *HER2* status was primarily assessed by immunohistochemistry on all cases. FISH assays were done on all 2+ and 3+ cases, and on some of the 1+/0 cases (as random spot testing for quality control).

2005–2010: *HER2* status was assessed by primary FISH testing on all cases. No immunohistochemical assessment for the HER2 protein was done in this period.

2011–2012: *HER2* status on primary breast cancer was assessed by a combined immunohistochemistry and FISH approach.

### Fluorescence in situ hybridization (FISH) for *HER2* status

2001–2012: For FISH analyses during the whole analyzed period, the *HER2* gene was tested by using a dual fluorescence kit (PathoVysion, Vysis, Abbott AG, Diagnostic Division Baar, Switzerland) containing the *HER2* gene (17q11.2 - q12, directly labeled with fluorescent spectrum orange) and *CEP17* (17p11.1 - q11.1, labeled with fluorescent spectrum green). Paraffin embedded sections of two micrometer thickness were used. Each case was accompanied by a corresponding hematoxylin and eosin (H&E) stain in order to identify the invasive tumor. All procedures were carried out by following the recommended protocol of the manufacturers.

2001–2010: Probe mixes were hybridized at 37°C between 14 and 20 h, washed in Rapid-Wash-Solution I at 73°C for 5 min, Rapid-Wash-Solution II and H_2_O for 7 min, air dried and counterstained with DAPI.

2011–2012: The whole procedure was carried out using the PathoVysion probes on the fully automated Leica Bond autostainer (Leica Biosystems, Nunningen, Switzerland).

Subsequently, the slides were evaluated by using an Olympus computer guided fluorescence microscope (BX61), the diagnostic areas from each case were documented by digital images.

### Guidelines for interpretation of in situ hybridization (FISH)

2001–2007: The FISH signals were interpreted in accordance with the FDA guidelines as follows: the number of signal copies and the ratios (*HER2/CEP17*) were calculated for each probe. Gene copies (>4) or cluster formations (small clusters ~ 6 copies, larger clusters ~ 12 copies) were defined as amplified. Ratios were defined as follows: ratio > 2.0 was set as amplified status; a ratio ≤ 2.0 was negative [12].

2008–2012: The ASCO-CAP guidelines were used for interpreting the signals in the FISH tests. Similarly to the previous period, the number of signal copies and the ratios (*HER2/CEP17*) were calculated for each probe Gene copies (> 6) or cluster formations (small clusters ~ 6 copies, larger clusters ~ 12 copies) were defined as amplified. Similarly, a ratio > 2.2 was set as amplified status; a ratio < 1.8 was negative, and a ratio of 1.8 - 2.2 was referred to as equivocal [3,13].

During the whole analyzed period (2001–2012), FISH *HER2* diagnostic service was covered by two board certified pathologists at the same time (together 4 pathologists over the 12 years), who acquired thorough expertise in *HER2* FISH assay interpretation.

The presence of aneusomy (polysomy) of chromosome 17 (> 4 copies) was documented in the histology reports during the whole analyzed period (2001–2012).

### Immunohistochemistry for HER2 status

2001–2004: The HER2 protein was determined by the FDA-approved antibody PATHWAY, anti-HER2/neu, clone CB11 (Ventana, Switzerland). The whole staining procedure (including pretreatment and staining) was conducted with the Ventana Benchmark semi-automated staining system using Ventana reagents for the entire procedure (including iVIEW DAB detection kit and the signal was enhanced using the amplification kit).

2011–2012: The HER2 protein was assessed by the FDA-approved antibody PATHWAY, anti-HER2/neu, clone 4B5 (ready to use without further dilution, concentration, Ventana, Switzerland). The whole procedure (including pretreatment and staining) was carried out using the 4B5 clone on the fully automated Leica Bond autostainer (Leica Biosystems, Nunningen, Switzerland). No signal-enhancement was used.

For immunohistochemistry, paraffin embedded sections of 2 micrometer thickness were used. HER2 IHC was interpreted during the whole period (2001–2004, 2011–2012) on light microscope without using digital imaging.

### Guidelines for interpretation of HER2 immunohistochemistry

2001–2004: The FDA approved DAKO guidelines were used for scoring as follows: 0 (no staining), 1+ (weak and incomplete membrane staining), 2+ (weak/moderate complete staining in more than 10% of the invasive tumor cells) or 3+ (strong complete homogenous membrane staining in more than 10% of the invasive tumor cells) [1,12]. During this period, HER2 IHC was assessed by all board certified staff members (approximately 15 pathologists) [2].

2011–2012: The ASCO-CAP guidelines were used for the interpretation of staining and HER2 protein expression was scored as 0 (no staining), 1+ (weak and incomplete membrane staining), 2+ (strong, complete membrane staining in less than 30% of the invasive tumor cells or weak/moderate heterogeneous complete staining in more than 10% of the invasive tumor cells) or 3+ (strong complete homogenous membrane staining in more than 30% of the invasive tumor cells) [3].

During this period, the vast majority of HER2 IHC (> 95%) was assessed by three breast pathologists.

### Guidelines for discordant cases

In case of discordant results between IHC and FISH, the HER2 IHC underwent a second look on light microscopy and in most cases IHC was repeated. If IHC did not change, the FISH result was considered as definitive. In borderline cases (IHC score 2+ and FISH ratio 1.8-2.2), we recommended a repeated HER2 status assessment on the excision specimen, or a repeated HER2 assessment on a further tumor block (with IHC and FISH).

### Statistics

The concordance-rate between IHC score 3+ and presence of *HER2* amplification by FISH in the two time periods 2001–2004 and 2011–2012 were compared for significant differences using Fisher’s exact probability test.

The same statistical analysis was performed for IHC score 0/1+ and the presence of *HER2* amplification by FISH in the two time periods 2001–2004 and 2011–2012 as well.

From 2005 to 2010, only FISH without IHC was performed, therefore not included in the statistics.

### Ethical considerations

The study was labeled as a quality control study and was conducted retrospectively as data analysis of an existing data bank without any additional experiment on human tissue. This study is a part of a previously approved project by the Ethical Committee of Canton Zurich (KEK-2012-553) and was conducted in a completely anonymized way.

## Results

### 2001–2004

We included only tumors in the concordance analysis, which underwent both IHC and FISH testing with interpretable assay results. Altogether 1062 breast cancer cases underwent double testing in these four years, 38 of them did not have interpretable clear signals in FISH testing or were equivocal (3.6%), these cases were excluded from the analysis, resulting in 1024 cases for this period.

**Figure 1:**
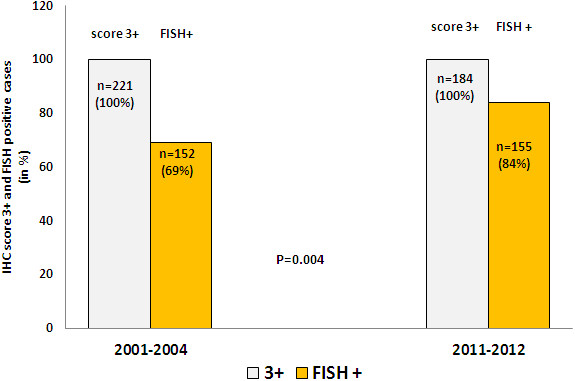
**Immunohistochemistry score 3+ absolute case number and concordance with FISH amplification between 2001–2004 and 2011–2012.** Double testing (IHC and FISH) was performed on all 3+ cases.

**Figure 2:**
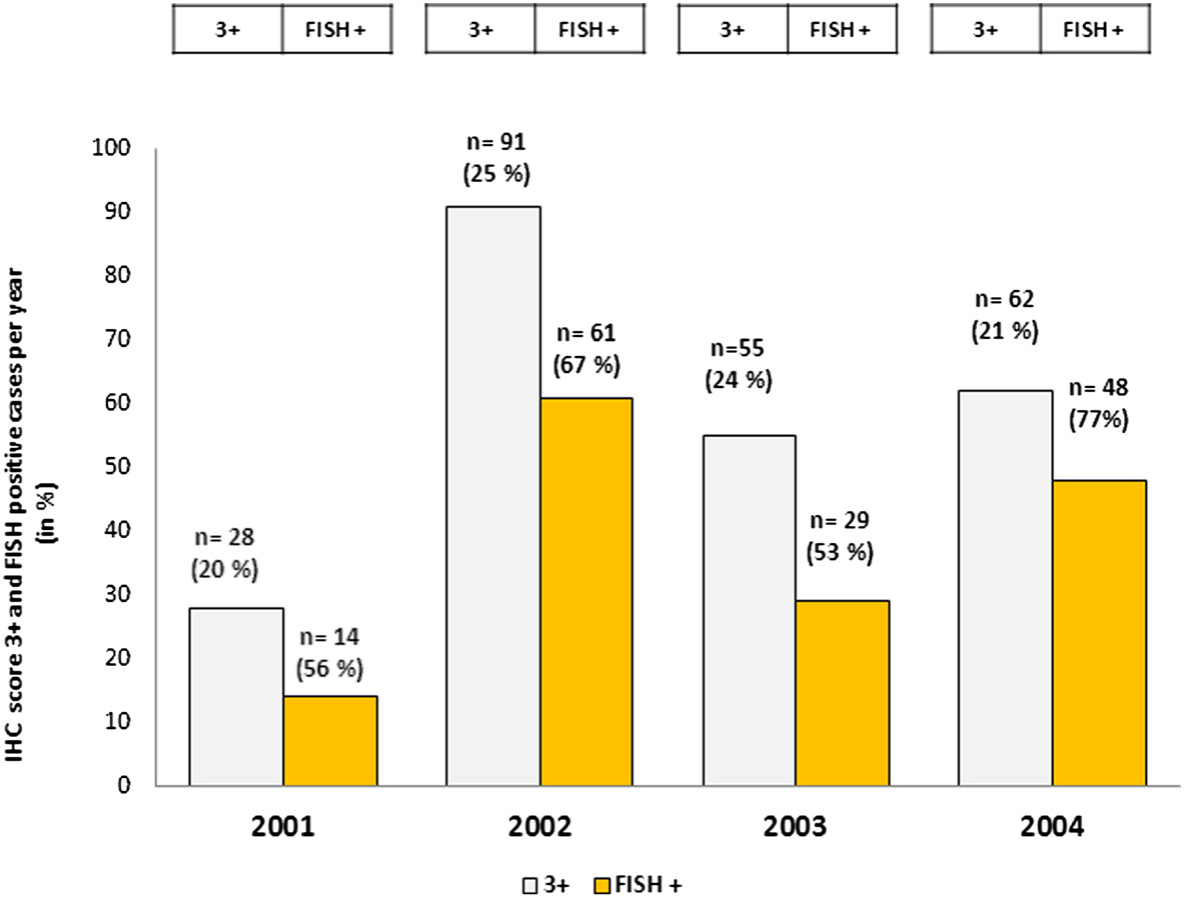
Variation IHC score 3+ and its concordance with FISH between 2001–2004.

**Figure 3:**
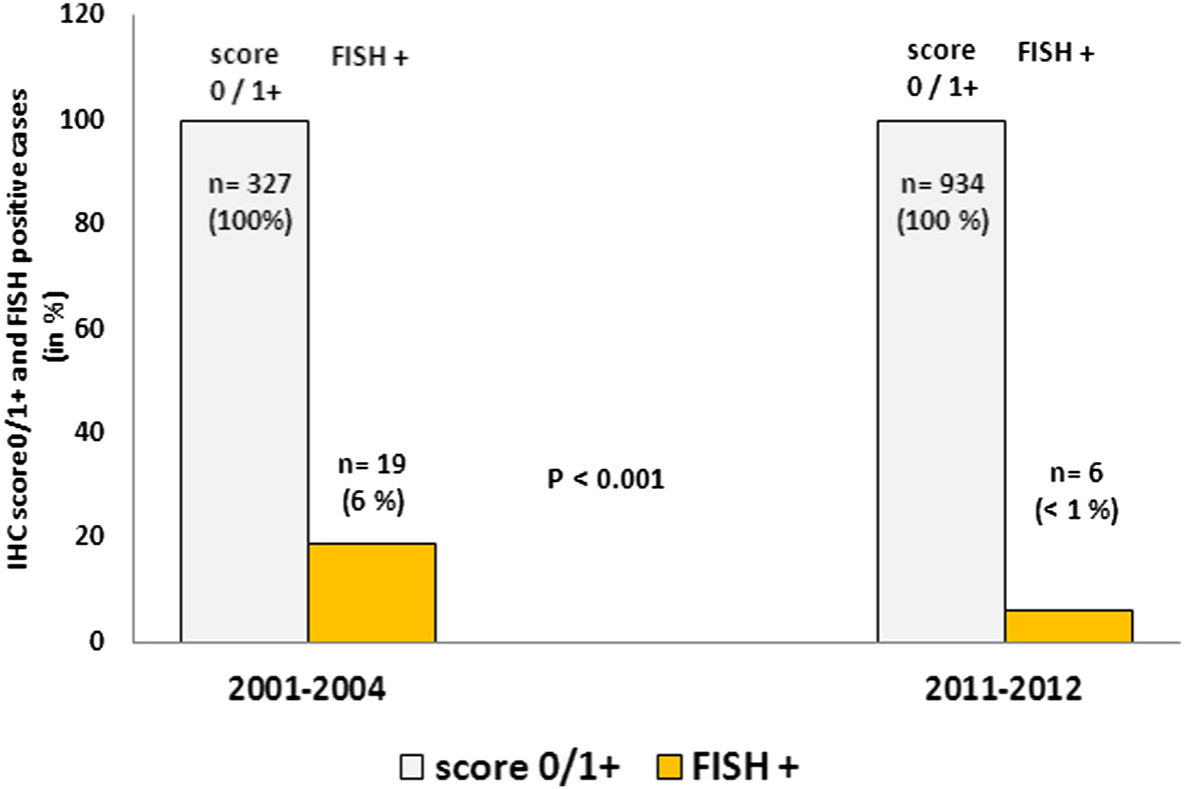
Results on double testing (IHC and FISH) on IHC 0/1+ cases and concordance with FISH amplification between 2001–2004 and 2011–2012.

22% of the 1024 cases were score IHC 3+ (221 of 1024 cases). Concordance between IHC 3+ and FISH was 69% (152 of 221 cases were amplified by FISH) (Table 1, Figure 1). There was a yearly variation on 3+ positive cases varying from 20 to 24%. Concordance between 3+ and FISH positivity showed a yearly variation from 53% to 77% (Figure 2). Among the analyzed score 0 and 1+ cases, 19 of 328 were amplified by FISH (6%), (Figure 3). 13% of IHC 2+ cases were positive in the FISH analysis.

**Table 1:** Concordance of test results in routine diagnostic HER2 assays in breast cancer

	**IHC**			
**FISH**	**0**	**1+**	**2+**	**3+**
Non amplified	35	273	413	69
	(90%)	(95%)	(87%)	(31%)
Amplified	4	15	63	152
	(10%)	(5%)	(13%)	(69%)
Total (n = 1024)	39	288	476	221
	(100%)	(100%)	(100%)	(100%)

Immunohistochemistry (IHC) and FISH between 2001 and 2004.

### 2005–2010

In this period, 4923 primary *HER2* FISH tests were conducted. 78 of them had no signals on FISH analysis, 2 cases were equivocal (1.6%). These 80 cases were excluded from the analysis, resulting in 4843 test assays for this period.

**Figure 4:**
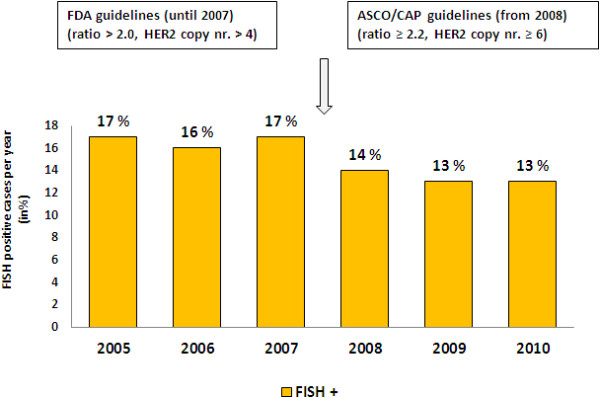
**Results HER2 amplification rate using FISH methodology as primary testing between 2005 and 2010.** Slight drop of FISH positive cases were observed after applying modified ASCO scoring criteria from 2008.

FISH amplification-rate was constant around 16% to 17% until the end of 2007. With the implementation of the modified ASCO/CAP guidelines in 2008, amplification-rate dropped to 13 to 14% and remained stable (Table 2, Figure 4).

**Table 2:** FISH results between 2005 and 2010

	**2005**	**2006**	**2007**	**2008**	**2009**	**2010**
Non-amplified	505	577	675	730	764	884
	(83%)	(84%)	(83%)	(86%)	(87%)	(87%)
Amplified	101	106	138	119	109	135
	(17%)	(16%)	(17%)	(14%)	(13%)	(13%)
Total (n = 4843)	606	683	813	849	873	1019
	(100%)	(100%)	(100%)	(100%)	(100%)	(100%)

### 2011–2012

In this period, a double testing (IHC and FISH) was conducted on all cases, altogether 1529 samples were analyzed. 7 of them had no signals on FISH, they were excluded from the analysis, resulting in 1522 *HER2* test assays.

12% of the cases were 3+ on IHC (184 of 1522 cases). 84% of 3+ cases were amplified by FISH. Among IHC score 0 and 1+ tumors by IHC, there were < 1% amplified tumors by FISH (6 cases of 934 cases). 5% of IHC 2+ cases were positive by FISH (Table 3, Figures 1 and 3).

**Table 3:** Concordance of test results in routine diagnostic HER2 assays in breast cancer

	**IHC**			
**FISH**	**0**	**1+**	**2+**	**3+**
Non amplified	361	567	384	29
	(> 99%)	(> 99%)	(95%)	(16%)
Amplified	3	3	20	155
	(< 1%)	(< 1%)	(5%)	(84%)
Total (n = 1522 )	364	570	404	184
	(100%)	(100%)	(100%)	(100%)

Immunohistochemistry (IHC) and FISH in 2011 and 2012.

Polysomy at FISH was detected in 99 of 7714 cases (1.2%) during the whole analyzed period (2001–2012).

### Concordance between FISH and IHC technologies

2001–2005: 19 cases (score 0 and 1+) were amplified and 69 cases (score 3+) were non-amplified in a total of 548 cases (after exclusion of 2+ cases), resulting in a concordance of 84% between IHC and FISH.

2011–2012: 6 cases (score 0 and 1+) were amplified and 29 cases (score 3+) were non-amplified in a total of 1118 cases (after exclusion of 2+ cases), resulting in a concordance of 97% between IHC and FISH.

### Statistical analysis

There was a significant different concordance-rate of IHC 3+ and the presence of FISH amplification between the periods 2001–2004 and 2011–2012 (p = 0.004). Similarly, there was also a significant different discordance of IHC 0/1+ and FISH amplification between the time periods 2001–2004 and 2001–2012 (p < 0.001).

## Discussion

In this study we demonstrate that concordance-rate between IHC and FISH *HER2* status in breast cancer significantly improved over 12 consecutive years and FISH only *HER2* testing resulted in a constant amplification-rate. We followed three different approaches in a single institution using IHC assays complemented with FISH, FISH only testing and double IHC/FISH testing in an exclusively diagnostic setting examining 7714 consecutive breast cancers.

Accuracy of HER2 testing in breast cancer is of enormous importance as a subsequent therapy decision for or against chemotherapy and Herceptin therapy is directly subjected to the HER2 status determined by the pathologists [3].

We could show in our consecutive diagnostic material over 12 years that concordance between immunohistochemistry and FISH technology in *HER2* status in breast cancer considerably improved since 2011 in our institution. Using immunohistochemistry as primary testing from 2001 to 2004 resulted in a substantial variation in score 3+ cases and in an unsatisfactory congruence to the complementary FISH testing (53% to 77%). On the other side, applying IHC and FISH (double testing) from 2011 on each case yielded stable IHC score 3+ and concordant FISH positive cases (84% of 3+ cases were FISH positive). Overall concordance between IHC and FISH technology was significantly improved from 84% (2001–2004) to 97% (2011–2012).

When using FISH methodology as primary testing between 2005 and 2010, we observed a constant positivity-rate from 16-17%, which slightly dropped to 13-14% after implementing the modified ASCO guidelines in 2008.

There are only a very few papers on large consecutive cohorts available in the literature, addressing the stability and changes of HER2 positivity over several years of testing [14-16]. Two large cohorts (n > 1000) described that HER2 positivity-rate dropped from 21-26% to 11-14% when tests were performed consecutively on primary breast cancers from 2003 and 2012 [14,16]. These two studies used one sole diagnostic approach: Bilous et al. followed primary IHC testing with complementary FISH assays in a multicenter setting, Vergara et al. conducted a double analysis (IHC and FISH) on each case in a single institution. We used three different diagnostic algorithms over 12 years in a single institution and could show that HER2 IHC positivity-rate was fairly variable, whereas FISH *HER2* positivity-rate remained constant over the years. Similarly to the data above, a slight decrease in HER2 positive cases were observed immediately after implementation of the modified ASCO/CAP guidelines 2008 also in our collective [3,14,16]. In another smaller cohort no differences were detected when HER2 positivity-rate was compared between 2003/2004 and 2008/2009, though the case number enrolled in this study was rather small (n < 1000) [15].

The decrease in HER2 positivity-rate is potentially to explain by the introduction of mammography screening with improved detection of early breast cancers [15,16]. Screen detection resulted in a shift to a different patient population with less HER2 positive cases in early breast cancer and with younger age at diagnosis [15,16]. Organized mammography screening in some cantons of Switzerland (started in the 1999’s) influenced disease stage distribution and had an impact on improved mortality [17]. This is clearly different in metastatic settings with no relevant change in HER2 positivity based on literature data [15,16].

The modified definition for cut-offs for *HER2/CEP17* ratios and for score 3+ IHC presumably also played a role in the improved congruency between IHC and FISH technology in our cohort [3]. Similarly to our own results, two larger studies also reported a drop in *HER2* positivity and simultaneously a better congruence after implementing the modified guidelines from 2008 [16,18]. Interestingly, the positivity-rate by FISH when using this assay as the primary test in our institution (n = 4843), resulted in a rather constant positivity-rate prior to and after applying the modified ASCO criteria. Additionally to strict adherence to the criteria mentioned above, it is important to note, that both IHC and FISH *HER2* assays are subjected to inter-observer variability without thorough expertise [2,13,19]. There are clear recommendations in the literature as to how many HER2 assays one pathologist should analyze per year and to limiting the number of pathologists who are involved in HER2 diagnostic service [2]. We believe that constant FISH *HER2* positivity over many consecutive years in our institution can be also connected to the small number of max 2 pathologists who exclusively interpreted FISH signals.

Standardization of technical issues as pre-analytical processing, time to fixation and the use of divergent fixatives are considered as further reasons for incongruent test results [6,20-22]. Based on established literature data it can be assumed that the way and duration of antigen retrieval and fixation can lead to inconsistent and incorrect IHC results [6,20-22]. FISH (and ISH) technology is less prone to fixation and laboratory errors than IHC tests, making FISH assay more reproducible if signal interpretation is carried out by experienced pathologists [6,12,13]. As reported and recommended by Tubbs and Hicks several years ago, the choice for FISH as primary *HER2* testing should be considered if discordance issues with IHC and FISH assays and/or inter-observer interpretation difficulties occur in the given institution [12,13]. Our choice for FISH only testing in our institution from 2005 was triggered exactly by these considerations. The poor performance and concordance between IHC and FISH assays 2001–2004 in our institution were potentially due to pre-analytical issues and laboratory as well as interpretational difficulties. We assume that the semi/manually conducted HER2 IHC stains with the highly sensitive and less specific CB11 antibody combined with signal interpretational difficulties in a large group of pathologist analyzing HER2 IHC stains, led to this poor performance [2,23,24]. It is well known that fixation can be significantly affect test results in IHC as variable fixation can make HER2 overexpressed protein undetectable (false negative) or native HER2 protein more detectable (equivocal or non-interpretable) [6,21,22].

Switching to FISH only methodology using one kit from Vysis-Abbott during the whole analyzed period yielded constant FISH amplification-rates over 6 years (2005–20120). The rate of FISH amplification was independent from fixation times even in the light of different external hospitals submitting breast cancer samples. Also interpretation difficulties of FISH signals was not a problem, as the number of pathologist, who interpreted the FISH *HER2* assays was limited to 2 experienced breast pathologists.

Therefore the considerable improvement in concordance between IHC and FISH assays is probably a result of the fully automated IHC and FISH procedures, a strict adherence to international guidelines (ASCO) and limited the number of pathologists responsible for the interpretation of *HER2* FISH and IHC results.

According to current ASCO guidelines there is no need for complementary FISH testing at IHC score 0/1+ cases [3]. We experienced FISH amplification in up to 6% of score 0/1+ samples in 2001–2004 with a considerable improvement of <1% in 2011–2012. Data on FISH amplification in score 0/1+ cases is inconsistent in the literature as FISH testing is not done systematically. In this IHC group, Gown reported a 0.8% FISH amplification-rate. In contrast, Martin et al. reported *HER2* amplification-rate by FISH in up to 14% in IHC score 0/1+ cases [25,26]. We assume that IHC score 0/1+ with FISH amplification represent most likely false negative results due to laboratory errors and fixation issues. Furthermore we believe that the improvement in this IHC group 2011–2012 is a consequence of automatic and standardized pre-analytical and interpretation issues, in a similar matter as the improvement in score 3+ cases from 2011.

Interestingly, the percentage of technically not interpretable or equivocal cases also showed a decrease over the last years [18]. In a large cohort, Middleton et al. reported a drop from 10 to 3.4%, which is quite similarly to our own observations (3.6% to 1.6%) [18].

The need for standardization and quality controlled HER2 testing were the subject already addressed in the early 2000s, when HER2 testing in breast cancer became a predictive marker for Herceptin therapy [1,23,24,27,28]. Useful practical recommendations became available from the early papers dealing with HER2 testing as to the use of commercially available and validated antibodies, internal validation, fixation issues, reporting and proper training of pathologists participating in HER2 testing [1,23,24,27,28].

Even from these early years there was a concern about incorrect HER2 results, the importance of all points mentioned above did not loose from their merit until now. Recent papers on HER2 testing add to the recommendations mentioned above, the need for pathology institutions to participate in external national or international quality assurance and proficiency programs together with develop national/international guidelines for HER2 testing [5,26,29-35]. During the last years, pitfalls in HER2 testing as polysomy and co-amplification of *HER2/CEP17* were explored and published, which pathologists must be also aware of, when reporting HER2 status in breast cancer [36-38].

## Conclusion

In summary, our study provides data on stable *HER2* positivity-rate determined by FISH technology on a large consecutive diagnostic breast cancer cohort and explores how concordance-rate between IHC and FISH technology can be improved by applying standardized analytical and pre-analytical procedures.

The question whether there is a best methodology as the gold standard for HER2 testing remains controversial [2,3,6]. Whatever methodology is used in the given pathology institution, standardized pre-analytical, analytical and scoring issues need to be guaranteed in order to provide accurate HER2 test results in the diagnostic setting.

## Abbreviations

IHC: Immunohistochemistry; FISH: Fluorescence in situ hybridization; HER2: Human epidermal growth factor receptor 2; ASCO: American society of clinical oncology; FDA: Food and drug administration.

## Competing interests

The author(s) declare that they have no competing interest.

## Authors’ contributions

ZV, AN: designed the paper, conducted HER2 FISH analysis, carried out analysis and interpretation of data and wrote the manuscript. CR, BP: conducted HER2 FISH analysis, carried out data interpretation and critically revised the manuscript. HM: designed the paper, helped to draft the paper and critically revised the manuscript. All authors read and approved the final manuscript.

## Pre-publication history

The pre-publication history for this paper can be accessed here:

http://www.biomedcentral.com/1471-2407/13/615/prepub
